# Insights into the Roles of Epigenetic Modifications in Ferroptosis

**DOI:** 10.3390/biology13020122

**Published:** 2024-02-15

**Authors:** Jinghua Kong, Hao Lyu, Qian Ouyang, Hao Shi, Rui Zhang, Shuai Xiao, Dong Guo, Qi Zhang, Xing-Zhen Chen, Cefan Zhou, Jingfeng Tang

**Affiliations:** 1 National “111” Center for Cellular Regulation and Molecular Pharmaceutics, Key Laboratory of Fermentation Engineering (Ministry of Education), Hubei University of Technology, Wuhan 430068, China; jinghua_hut@163.com (J.K.); haolyu@hbut.edu.cn (H.L.); oyq436670@163.com (Q.O.); 13971706429@163.com (H.S.); 2 Cooperative Innovation Center of Industrial Fermentation (Ministry of Education & Hubei Province), Hubei Key Laboratory of Industrial Microbiology, Hubei University of Technology, Wuhan 430068, China; zhangrui1987@hbut.edu.cn (R.Z.); xiaoshuai825@hotmail.com (S.X.); jk1103@whu.edu.cn (D.G.); zhangqi@hbut.edu.cn (Q.Z.); 3 Membrane Protein Disease Research Group, Department of Physiology, Faculty of Medicine and Dentistry, University of Alberta, Edmonton, AB T6G2R3, Canada; xzchen@ualberta.ca

**Keywords:** ferroptosis, epigenetic modification, DNA methylation, RNA methylation, non-coding RNA, histone modification

## Abstract

**Simple Summary:**

Since ferroptosis was proposed in 2012, it has been a popular field of study for researchers. So far, the regulatory mechanism of ferroptosis involves many aspects, such as DNA, RNA, and proteins. Accumulating studies have shown that ferroptosis and epigenetic modifications are crucial in multiple diseases. This review provides information on resistance systems to ferroptosis and advanced studies of epigenetic modifications in DNA methylation, RNA methylation, non-coding RNAs, and histone modifications through regulating ferroptosis in cancer and other diseases. A summary of the targets of epigenetic modifications regulating ferroptosis could help identify new prognostic indicators in human diseases and provide potential therapeutic strategies for these diseases.

**Abstract:**

Ferroptosis is a non-apoptotic mode of cell death driven by membrane lipid peroxidation and is characterized by elevated intracellular levels of Fe^2+^, ROS, and lipid peroxidation. Studies have shown that ferroptosis is related to the development of multiple diseases, such as cancer, neurodegenerative diseases, and acute myeloid leukemia. Ferroptosis plays a dual role in the occurrence and development of these diseases. Ferroptosis mainly involves iron metabolism, ROS, and lipid metabolism. Various mechanisms, including epigenetic regulation, have been reported to be deeply involved in ferroptosis. Abnormal epigenetic modifications have been reported to promote tumor onset or other diseases and resistance to chemotherapy drugs. In recent years, diversified studies have shown that epigenetic modification is involved in ferroptosis. In this review, we reviewed the current resistance system of ferroptosis and the research progress of epigenetic modification, such as DNA methylation, RNA methylation, non-coding RNAs, and histone modification in cancer and other diseases by regulating ferroptosis.

## 1. Introduction

Ferroptosis is a non-apoptotic cell death mode with the characteristics of mitochondrial membrane shrinkage and increased mitochondrial membrane density [1]. Ferroptosis is not characterized by the morphological features of typical necrosis, such as the swelling of organelles or the rupture of cell membranes; nor is it characterized by traditional apoptosis, such as cell shrinkage, chromatin condensation, the formation of apoptotic vesicles, or cytoskeletal disintegration. In contrast to autophagy, ferroptosis does not form a bilayer membrane structure [2]. The occurrence of ferroptosis is regulated by cystine depletion and massive lipid peroxidation, dependent on reactive oxygen species (ROS), polyunsaturated fatty acid (PUFA)-containing phospholipids (PUFA-PLs), and transition metal iron. The classical mechanism for lipid peroxidation scavenging is the glutathione (GSH)–glutathione peroxidase 4 (GPX4) axis, which was found to reduce lipid peroxides to lipid alcohols [3]. Jiang et al. reported that the tumor protein p53 promotes ferroptosis through the activation and integration of PUFAs, caused by the inhibition of solute carrier family 7 member 11 (SLC7A11), Acyl-CoA synthetase long-chain family member 4 (ACSL4), and lysophosphatidylcholine acyltransferase 3 (LPCAT3) [4,5]. A recent study has shown that the ferroptosis suppressor protein 1 (FSP1) inhibitor icFSP1 increases the level of cellular ferroptosis by driving the phase separation of FSP1, leading to an increased susceptibility of cancer cells to ferroptosis sensitization [6]. In another study, vitamin K inhibited the toxic effects of FSP1-dependent ferroptosis on cells [7]. Vitamin E was shown to rescue the Gpx4-deficient hematopoietic stem and progenitor cells from ferroptosis in vitro [8]. In addition to the classical SLC7A11-GPX4 axis, four additional mechanisms have been identified to be involved in the regulation of ferroptosis: the FSP1/ubiquinone (CoQ10)/NADPH pathway [9], the GTP cyclohydrolysate 1 (GCH1)/tetrahydrobiopterin (BH4) pathway [10], the dihydroorotate dehydrogenase (DHODH) pathway [11], and the O-acyltransferase 1/2 (MBOAT1/2)-monounsaturated fatty acid (MUFA) system [12]. It has been shown that ferroptosis is widespread in humans, mammals, plants, protists, and fungi [13]. Hence, we compared the mechanisms by which ferroptosis takes place in protists, plants, animals, and microbes (Table 1).

**Figure 1:**
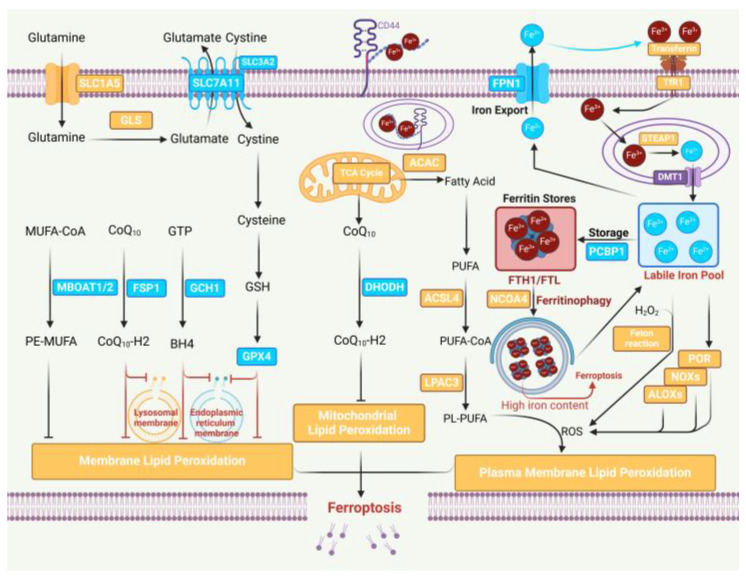
The figure is created via the biorender application, (BioRender.com, accessed on 24 December 2023). Core mechanisms of ferroptosis. The iron-dependent peroxidation of PUFA-PLs is central to ferroptosis. At least four ferroptosis-resistant systems have been identified in cells, including the GPX4/xCT system, the FSP1/CoQH2 system, the DHODH/CoQH2 system, and the GCH1/BH4 system, which prevent lipid peroxidation in different subcellular localizations, thereby protecting cells from ferroptosis. ACSL4 and LPCAT3 are engaged in the synthesis of PUFA-PLs. Iron initiates the Fenton reaction with H_2_O_2_ and is an important cofactor for ALOXs (arachidonate lipoxygenases) and POR (cytochrome P450 reductase), promoting lipid peroxidation. Once the induction of ferroptosis significantly exceeds the detoxification capacity of the ferroptosis-resistant system, the accumulation of lipid peroxides on cell membranes leads to membrane rupture and triggers ferroptosis.

Iron metabolism, ROS, and lipid metabolism are critical for executing ferroptosis (Figure 1). The process of iron metabolism in ferroptosis comprises iron uptake, storage, and degradation [19], and the aberrant activation of each process may accompany the execution of ferroptosis. Iron is normally imported into cells via the transferrin/transferrin receptor (Tf-TfR) system [20]. Excess intracellular iron is stored by ferritin, which is composed of two subunits: ferritin light chain (FTL) and ferritin heavy chain 1 (FTH1). When ferritin binds to nuclear receptor coactivator 4 (NCOA4), it is transported to the lysosome for autophagic degradation, and the iron stored in ferritin is released into the labile iron pool, which regulates iron levels and determines sensitivity to ferroptosis [21,22]. Iron efflux is also a crucial pathway that affects ferroptosis. The exocytosis of free Fe^2+^ and the oxidation of Fe^2+^ to Fe^3+^ are both facilitated by the iron transport protein 1 (FPN1). ROS are produced as a result of normal cellular metabolic processes, including the generation of superoxide anions (O_2_^−^), hydroxyl radicals (OH^−^), hydrogen peroxide (H_2_O_2_), and singlet oxygen (^1^O_2_). The major cellular sources of ROS are the mitochondrial electron transport chain (mETC) and NADPH oxidases (NOXs) in the cell membrane [23]. The NOX family has seven members: NOX1, cytochrome B-245 β-chain (CYBB/NOX2), NOX3, NOX4, NOX5, dioxygenase 1 (DUOX1), and dioxygenase 2 (DUOX2). They are part of a membrane-bound enzyme complex that, along with other proteins, aids in the transfer of electrons across the cell membrane, leading to the production of superoxide and other downstream ROS [24]. In previous research on ferroptosis, the accumulation of ferroptosis-associated ROS has emerged as an important indicator of whether ferroptosis has occurred. The peroxidation of cell membrane lipids is a prerequisite for ferroptosis, which is mediated by lipid metabolism. PUFAs are required for membrane lipid peroxidation and its oxidation, carried out within the membrane, may result in oxidative reactions propagating through the membrane [25]. Large amounts of uncontrolled membrane lipid peroxidation can lead to organelle dysfunction and a loss of plasma membrane integrity, resulting in the ferroptosis of cells [26,27]. ACSL4 [28], LPCAT3 [29], cytoplasmic-type phospholipase A2 (cPLA2α) [30], and Ca^2+^-independent phospholipase A2 (iPLA2β) [31] were identified as ferroptosis modulators involved in lipid peroxidation [32]. The MBOAT2 functions as a lysophospholipid acyltransferase (LPLAT) that dopes MUFAs into lyso-phosphatidylethanolamine (Lyso-PE), increasing the levels of cellular PE-MUFAs, decreasing the levels of cellular PE-PUFAs, and ultimately inhibiting the production of lipid peroxides to inhibit ferroptosis. A recent study has shown that 7-dehydrocholesterol (7-DHC) effectively prevents phospholipid peroxidation and thus plays a protective role in regulating ferroptosis [33,34].

## 2. Epigenetic Modification

Although the regulatory mechanisms of several critical factors in the development of ferroptosis have been reported, the epigenetic regulation in the development of ferroptosis needs to be better understood. Epigenetic modification regulates gene expression by altering DNA and proteins on chromosomes through chemical modifications that affect gene expression [35]. Epigenetic modifications can affect multiple levels of gene expression, including transcription, splicing, stability, translation, nucleosome assembly, and chromatin structure. As a result, they can influence both physiological and pathological cellular processes, as well as the phenotype of offspring [36,37]. Epigenetic modifications determine how DNA is translated, the strict regulation of DNA structure, and the consequent control of the expression of specific genes at specific times, including DNA methylation, RNA methylation, non-coding RNAs, histone modifications, and so on [38]. Epigenetic modifications are often involved in the development of multiple diseases and tumors [39,40,41]. Emerging studies have confirmed that aberrant DNA methylation is closely related to tumors and is a common epigenetic phenomenon in the process of tumor development, including the silencing of tumor suppressor genes or the inactivation of damage repair genes due to elevated levels of methylation of CpG islands in the promoter regions of specific genes [42,43]. Other epigenetic modifications, such as RNA methylation, non-coding RNAs, and histone modifications, also play crucial regulatory roles in diseases and tumors, and an understanding of the full spectrum of epigenetic modifications may provide new ideas for the treatment of cancer and other diseases.

Accumulating studies have shown that epigenetic modifications can transcriptionally and translationally determine the susceptibility of cancer cells to ferroptosis and correlate with the progression of a wide range of diseases, such as osteoarthritis [44], pulmonary arterial hypertension [45], spinal cord injury [46], and sickle cell disease [47]. Herein, we review the latest research on epigenetic modifications regulating ferroptosis in cancer and other diseases, contributing to identifying new prognostic indicators and therapeutic targets in tumors and other diseases.

## 3. DNA Methylation Regulates Ferroptosis

DNA methylation is the most common modification that regulates gene expression. It was discovered in the 1970s by Holliday and Pugh as a major source of epigenetic inheritance [48]. 5-methylcytosine (5-mC) is the most common modification in DNA, placing a methyl group on the fifth carbon atom of the nucleotide cytosine. In mammals, the methylation modification of DNA is mediated by DNA methyltransferases (DNMTs), such as DNMT1, DNMT3A, and DNMT3B. This modification generally has a negative effect on gene expression [49,50].

Emerging studies suggest that DNA methylation is primarily involved in ferroptosis and that targeting the induction of ferroptosis may be a new avenue for cancer treatment. A recent study showed that glycine-enhanced GPX4 promoter methylation catalyzed by DNMT1, DNMT3A, and DNMT3B induces ferroptosis in rheumatoid arthritis [51]. In addition, DNA dioxygenase ten-eleven translocation 2 (TET2) is an important demethylase that inhibits ferroptosis through GPX4 promoter demethylation in airway epithelial cells [52]. A recent study found that FSP1 expression is repressed through promoter hypermethylation, which leads to increased ferroptosis sensitivity by the GSH-GPX4 axis in acute lymphoblastic leukemia cell lines [53]. The DNA methylation inhibitor 5-azacitidine (5-Aza) inhibits ferroptosis by increasing CDH1 expression. [54]. The 5-Aza decreased the methylation level of protocadherin beta 14 (PCDHB14), which induces ferroptosis in hepatocellular carcinoma cells. The upregulation of PCDHB14 mediated by p53 promotes the ubiquitination of p65 mediated by E3 ubiquitin ligase RNF182 and accelerates its degradation, resulting in the inhibition of p65-mediated SLC7A11 transcriptional expression [55]. The abnormality pattern of 5-mC is often closely associated with cancer development, and recent studies have shown that ferroptosis can influence the development of various cancers [32]. Lymphocyte-specific hemolysin (LSH) is a reader of 5-hmC [56]. It interacts with WDR domain protein 76 (WDR76) to inhibit ferroptosis by promoting the expression of glucose transporter 1 (GLUT1), stearoyl-CoA desaturase 1 (SCD1), and fatty acid desaturase 2 (FADS2) [57]. A recent study has found that high levels of methylation of the solute carrier family 2 member 1 (SLC2A1) gene in colorectal cancer patients are positively associated with the inhibition of ferroptosis and immunosuppression. Colorectal cancer patients with high SLC2A1 expression have a poor prognosis. These findings suggest that SLC2A1 may play a role in the tumor immunomodulation of colorectal cancer by regulating ferroptosis [58]. DNA methylation can affect ferroptosis through classical or other pathways, which has significant implications for preventing excessive ferroptosis and developing technologies to conditionally control it.

## 4. RNA Methylation Regulates Ferroptosis

RNA carries a wide range of chemical modifications, such as N6-methyladenosine (m6A), N1-methyladenosine (m1A), and cytosine hydroxylation (m5C), which play essential roles in the regulation of gene expression. In eukaryotes, m6A accounts for about 80% of RNA methylation modifications and is marked by m6A methylase. Jia et al. found that the m6A modification is reversible and identifies the m6A demethylase fat and obesity-related genes (FTO) [59], and another m6A demethylase AlkB homolog 5 (ALKBH5) is also discovered [60]. Reading proteins act on RNA by recognizing m6A marks and include YT521-B homology (YTH) domain proteins as well as insulin-like growth factor 2 mRNA binding protein (IGF2BP) [61]. The m6A methylases are called “writers”, such as METTL3, METTL14, and WTAP; demethylases are called “erasers”, such as FTO and ALKBH5; reading proteins are called “readers”, including YTHDC1, YTHDC2, YTHDF1, YTHDF2, and IGF2BP1 [62,63]. The “reader” mediates the translation, stability, splicing, and nuclear export of mRNAs by recognizing m6A marks. In addition, the latest study has shown that exon junction complexes are “suppressors” of m6A and inhibit the functions of the “writer” to regulate the regional selectivity of m6A, which determines the specificity of the overall distribution of m6A epistasis [64]. The m6A modification is a novel post-transcriptional regulatory mechanism that has been shown to play an important role in ferroptosis [65,66].

**Figure 2:**
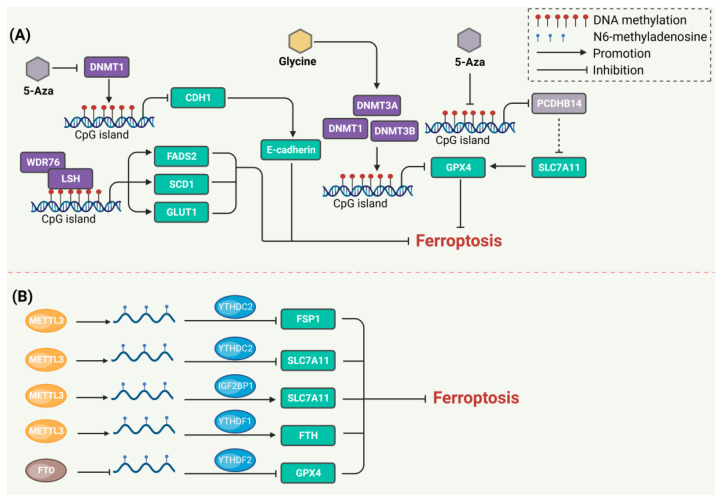
The regulation of ferroptosis by DNA and RNA methylation. (**A**) DNA methylation in ferroptosis. LSH interacts with WDR76 and inhibits ferroptosis through affecting the DNA methylation of FADS2, SCD1, and GLUT1. DNMTs including DNMT1, DNMT3A, and DNMT3B usually inhibit the expression of target genes to regulate ferroptosis. Inversely, the DNMT inhibitor 5-Aza antagonizes the biological function of DNMTs. (**B**) RNA m6A modification in ferroptosis. METTL3 increases the m6A modification of SLC7A11 and FSP1 RNA. YTHDC2 acts as a SLC7A11 and FSP1 mRNA reader, leading to mRNA decay to induce ferroptosis. IGF2BP1 acts as a SLC7A11 mRNA reader and increases its translation to inhibit ferroptosis. FTO inhibits the m6A modification of GPX4 mRNA. YTHDF2 acts as a GPX4 mRNA reader, resulting in mRNA decay to induce ferroptosis. The dotted line represents indirect regulation.

Emerging studies have shown that YTHDC2 expression was low in lung cancer and that it induced ferroptosis by inhibiting the expression of SLC7A11 through m6A modification [67,68]. NF-κB-activating protein (NKAP) positively regulates SLC7A11 expression, whereas the regulation can be reversed by m6A inhibitor cyclic leucine and the knockdown of METTL3. NKAP is a possible inhibitor of ferroptosis that protects glioblastoma cells from undergoing ferroptosis by enhancing the splicing and maturation of SLC7A11 mRNA through m6A modification [69]. ALKBH5 also inhibits tumor growth in colorectal cancer in vitro. Mechanistically, ALKBH5 promotes SLC7A11 mRNA decay to induce ferroptosis [70]. Targeting the m6A-ferroptosis axis would be a very promising therapeutic strategy. A recent study found that the expression of SLC7A11 increased in hepatoblastoma due to the METTL3-IGF2BP axis modifying and recognizing the SLC7A11 mRNA to stabilize it [71]. Ferroptosis plays a role in several cardiovascular diseases, and targeting ferroptosis or m6A modification may be a promising strategy. METTL3 promotes ferroptosis in human aortic smooth muscle cells by inhibiting the expression of SLC7A11 and FSP1 [72]. A recent study found that targeting AKT significantly induced GPX4-dependent ferroptosis and inhibited the growth of colorectal cancer cells. AKT inhibitors elevate GPX4 m6A levels and promote YTHDF2-mediated GPX4 mRNA decay by reducing FTO [73]. A previous study found that METTL3 promotes the FTH m6A methylation and enhances its mRNA stability in a YTHDF1-dependent manner, with YTHDF1 inhibiting ferroptosis by upregulating FTH in lung cancer [74,75,76,77]. Other RNA methylation modifications, such as m1A and m5C, have also been reported to be involved in the post-transcriptional modification of ferroptosis-related genes [78,79]. Therefore, more RNA methylation targets that regulate ferroptosis need to be discovered in further studies to provide insights for clinical treatment (Figure 2).

## 5. NcRNAs Regulating Ferroptosis

Non-coding RNAs (NcRNAs) play a significant role in regulating cellular processes. NcRNAs are non-coding transcripts with limited protein-coding potential and exert essential cellular functions through different molecular mechanisms [80]. In a broad sense, they can be subdivided into short and long ncRNAs and their biological functions, including short-stranded microRNAs (miRNAs), long-stranded non-coding RNAs (lncRNAs), and circular RNAs (circRNAs) [81,82].

MiRNAs are small ncRNAs, about 22 nucleotides long, widely found in eukaryotes and conserved throughout evolution. Single miRNAs can regulate multiple target genes, and multiple miRNAs can also regulate the same gene. The main function of miRNAs is the post-transcriptional regulation of gene expression by binding to complementary target mRNA sequences, leading to translational repression or mRNA degradation that halts protein synthesis [83,84]. It has been shown that miRNAs may also induce gene expression by binding to target sequences and acting as translation activators [85]. Although miRNAs were not known over 30 years ago, they now regulate the expression of over 60% of protein coding genes. [86]. Exosome is a general term for many extracellular vesicles, a type of vesicle actively secreted by the cell and encapsulated by a phospholipid bilayer in which miRNAs are encapsulated. Almost every cell or tissue in the animal body can release exosomes externally, and miRNAs can be transported to various target cells and target organs through exosomal vehicles [87,88]. An abnormal expression of miRNA is often correlated with cardiovascular, autoimmune, infectious, and neurodegenerative diseases [89,90]. MiRNAs are also involved in cancer development, acting as tumor suppressors or oncogenes [91]. Emerging studies have shown that miRNAs participate in the critical regulation of ferroptosis in cancer, but the mechanism of their regulatory role needs to be further investigated.

LncRNAs are a class of heterogeneous ncRNAs that are more than 200 nucleotides in length. They are similar to mRNAs in transcriptional and post-transcriptional mechanisms [92]. According to recent studies, LncRNAs play a crucial role in regulating cellular processes by interacting with other molecules such as DNA, RNA, and proteins. This finding sheds light on the important functions of lncRNAs in cellular processes [93,94]. H19 [95] and Xist [96] were first discovered in lncRNAs in the 1980s and 1990s. In the beginning of the 21st century, when the characteristics of ncRNAs started to exceed protein-coding genes, the role of lncRNAs began to be noticed [97]. Aberrant lncRNA expression involves all hallmarks of cancer, including sustained angiogenesis and dysregulated cellular metabolism [98,99]. In addition, there is growing evidence for their importance in regulating ferroptosis.

**Figure 3:**
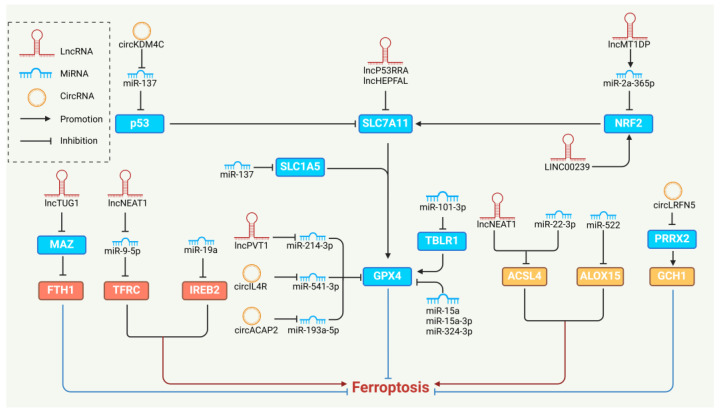
The regulation of ferroptosis by NcRNAs. NcRNAs target metabolizable molecules such as ACSL4 and ALOX15 in lipid metabolism to regulate ferroptosis. NcRNAs regulate ferroptosis in classical and non-classical signaling pathways, such as the p53/NRF2-SLC7A11-GPX4 axis and GCH1. NcRNAs target iron-related proteins such as FTH1 [104], TFRC [105], and IREB2 [106], and regulate ferroptosis in iron metabolism.

CircRNAs are single-stranded, covalently closed ncRNA molecules with different characteristics from other ncRNAs [100]. Initially, they were thought to be simply splicing disturbances and procedural errors produced by irregular splicing. Thus, their biological relevance was ignored. CircRNAs are rich in miRNA-binding sites and act as miRNA sponges, competing with target mRNAs for binding miRNAs, thereby inhibiting the degradation of target mRNAs [101,102]. CircRNAs play essential roles in various biological functions, such as miRNA sponges, transcriptional regulators, and RNA-binding proteins. CircRNAs are associated with developing many normal and pathological cellular processes and diseases [103], and it has been shown that they are implicated in various ferroptosis regulatory mechanisms (Figure 3).

### 5.1. MiRNAs and Ferroptosis

The long-chain non-coding RNA lncPVT1 directly binds to miR-214-3p to inhibit its expression, whereas miR-214-3p promotes ferroptosis by targeting the degradation of GPX4 [107]. The expression of miR-101-3p was downregulated in lung cancer. MiR-101-3p promotes ferroptosis by targeting TBL1-related protein 1 (TBLR1) to downregulate GPX4 and upregulate prostaglandin-endoperoxide synthase 2 (PTGS2). By developing nanomedicines, miR-101-3p can be delivered to tumor cells in vivo for ferroptosis restoration and ultimately inhibit tumor proliferation [108]. MiR-324-3p was significantly downregulated in lung cancer cell lines compared to normal cells. MiR-324-3p induced ferroptosis and enhanced the sensitivity of cisplatin to ferroptosis via targeted GPX4 [109]. MiR-324-3p was upregulated by metformin in breast cancer cell lines and downregulated GPX4 to induce ferroptosis [110]. In colorectal cancer, miR-15a-3p promotes ferroptosis by inhibiting GPX4 and increasing the abundance of ROS, Fe^2+^, and MDA [111]. MiR-15a inhibited GPX4 expression in pancreatic cancer, leading to increased intracellular levels of lactate dehydrogenase, Fe^2+^, and ROS, thereby promoting ferroptosis. In conclusion, the induction of ferroptosis by these miRNAs through the regulation of GPX4 provides a basis for investigating therapeutic strategies for various cancers [112].

Exosomes play a crucial role in the communication between proximal and distal organs, regulating diseases through paracrine mechanisms. Cancer-associated fibroblasts inhibit ferroptosis in gastric cancer cells by targeting ALOX15 via the exosomal secretion of miR-522 and preventing lipid ROS accumulation [113]. Emerging studies in melanoma cells showed that miR-137 inhibited lipid peroxidation and iron accumulation by directly targeting solute carrier family 1 member 5 (SLC1A5). The non-Xc-system member SLC1A5 is a neutral amino acid transport protein for alanine, serine, cysteine, and glutamine [114,115]. Recent studies have shown that miR-22-3P expression is significantly upregulated in cardiomyocytes and plasma exosomes from mice with chronic myocardial infarction and patients with heart failure. The overexpression of miR-22-3p abolished erastin-induced ferroptosis in vitro. The ACSL4 is a crucial gene for fatty acid metabolism and a target gene of miR-22-3p in tumor cells. Myocardial infarction (MI) inhibits erastin-induced ferroptosis by releasing miR-22-3p-enriched exosomes derived from cardiomyocytes [116]. Thus, targeting exosome-mediated cardiomyocyte/tumor pathology communication may provide a new avenue for antitumor therapy based on ferroptosis. Previous studies have shown that iron-responsive element-binding protein 2 (IREB2) has been identified as an inducer of ferroptosis. MiR-19a represses ferroptosis by inhibiting IREB2 in colorectal cancer [106]. In summary, miRNAs can regulate ferroptosis by degrading inducers or inhibitors of ferroptosis, and exploring drugs that can target these miRNAs will be a new direction for synergistic tumor therapy.

### 5.2. LncRNAs and Ferroptosis

It was shown that lncHEPFAL expression was reduced in hepatocellular carcinoma tissues. The results indicate that lncHEPFAL promotes ferroptosis by mediating the ubiquitinated-dependent degradation of SLC7A11 and subsequently increasing lipid ROS and Fe^2+^ [117]. The tumor suppressor lncP53RRA is lowly expressed in lung and liver cancer [118]. Emerging studies in lung cancer have shown that lncP53RRA interacts with Ras GTPase-activated protein-binding protein 1 (G3BP1) in the cytoplasm. lncP53RRA decreased p53 binding to G3BP1 in the cytoplasm and increased the accumulation of p53 in the nucleus to promote SLC7A11 transcription and inhibit ferroptosis [119]. LncMT1DP regulates erastin-induced ferroptosis by stabilizing miR-2a-365p and inhibiting NF-E2 p45-related factor 2(NRF2) MT1DP which induces ferroptosis in non-small-cell lung cancer by increasing the abundance of ROS, MDA, and Fe^2+^. To enhance drug efficacy, folate (FA)-modified liposome (FA-LP) nanoparticles containing erastin and lncRNA-MT1DP (E/M@FA-LPs) increased sensitivity to erastin-induced ferroptosis by delivering erastin and MT1DP [120]. Tumor resistance or self-protective mechanisms limit the treatment of tumors, and the combination of non-coding RNAs with tumor therapy-related drugs will be an effective means to improve the therapeutic effect.

LINC00239 is an abnormally highly expressed tumor-promoting factor in colorectal cancer tissues and promotes tumor development by decreasing erastin- and RSL3-induced ferroptosis. LINC00239 inhibits NRF2 ubiquitination and increases NRF2 protein stability by interacting with the Kelch structural domain of Keap1 [121], thereby inhibiting ferroptosis. Nuclear enriched transcript 1 (NEAT1) is an oncogenic lncRNA distributed around the nucleus that affects cancer cell proliferation, cell cycle, invasion, migration, and apoptosis. NEAT1 could bind to ACSL4 mRNA, decreasing ACSL4 and inhibiting ferroptosis. NEAT1 does not significantly affect the expression of other ferroptosis factors under erastin-induced conditions, such as SLC7A11, GPX4, and TfR1, which suggests that its inhibitory effect on ferroptosis is mediated exclusively through ACSL4 [122]. In another study, lncRNAs were critical mediators in regulating iron metabolism during ferroptosis. lncNEAT1 increased cellular iron concentration, while lncRNA PR11-89 decreased cellular iron concentration to regulate ferroptosis. The former sponge miR-9-5p upregulated the expression of TFRC and GOT1, and the latter sponge miR-129-5p upregulated the expression of PROM2 [105,123]. Dihydroartemisinin (DHA) is a semi-synthetic derivative of artemisinin. Studies have shown that it has anti-glioma activity by inducing apoptosis and inhibiting the proliferation, migration, and invasion of glioma cells. Recent studies have shown that DHA can exert antitumor effects by inducing ferroptosis in glioma cells. However, the mechanisms of attenuated ferroptosis have also been demonstrated in DHA-treated glioma cells [104]. The study revealed that the downregulation of lncRNA TUG1 in DHA-treated glioma cells directly led to the upregulation of MYC-associated zinc finger protein (MAZ), which promotes FTH1 to block ferroptosis. Emerging studies suggest that lncRNAs can affect ferroptosis by targeting ferroptosis-associated transcription factors or regulators. Targeting these lncRNAs to affect ferroptosis is a potential therapeutic strategy to enhance antitumor effects.

### 5.3. CircRNAs and Ferroptosis

CircKDM4C was significantly downregulated in patients with acute myeloid leukemia (AML). CircKDM4C in AML cell lines promotes ferroptosis and inhibits cell proliferation, migration, and invasion. CircKDM4C inhibits the expression of hsa-let-7b5p as a sponge in AML cell lines, resulting in the upregulation of p53, which is the target gene of hsa-let-7b-5p.The transcription of SLC7A11 is inhibited by p53, which promotes ferroptosis [124]. CircIL4R is highly expressed in hepatocellular carcinoma and promotes tumorigenesis caused by regulating the miR-541-3p/GPX4 axis to inhibit ferroptosis [125]. CircLRFN5 expression is downregulated in glioblastoma, and the overexpression of CircLRFN5 inhibits the survival and proliferation of glioma stem cells (GSCs) as well as tumorigenesis by inducing ferroptosis [126]. CircLRFN5 binds to the transcription factor pairing-related homology box 2 (PRRX2), which promotes the degradation of PRRX2 via the ubiquitin-proteasome system and contributes to the reduction of GCH1, which is a key factor in promoting BH4 production. Targeting circLRFN5 to induce ferroptosis would be a promising therapeutic option for glioblastoma. CircRNA ACAP2 inhibits ferroptosis during cervical cancer progression via the miR-193a-5p/GPX4 axis [127]. CircACAP2 directly interacts with miR-193a-5p targeted GPX4 as a competitive RNA (ceRNA) in cervical cancer cells. Meanwhile, CircACAP2 inhibited the expression of miR-193a-5p by sponge-wrapping it, thereby promoting GPX4 expression in cervical cancer cells. CircRNAs are directly or indirectly involved in amino acid metabolism, lipid metabolism, and iron metabolism in ferroptosis. Further investigation is required to determine whether circRNAs have an exact mechanism of action in different cells and tissues, as they are a promising therapeutic target.

## 6. Histone Modifications Regulating Ferroptosis

**Figure 4:**
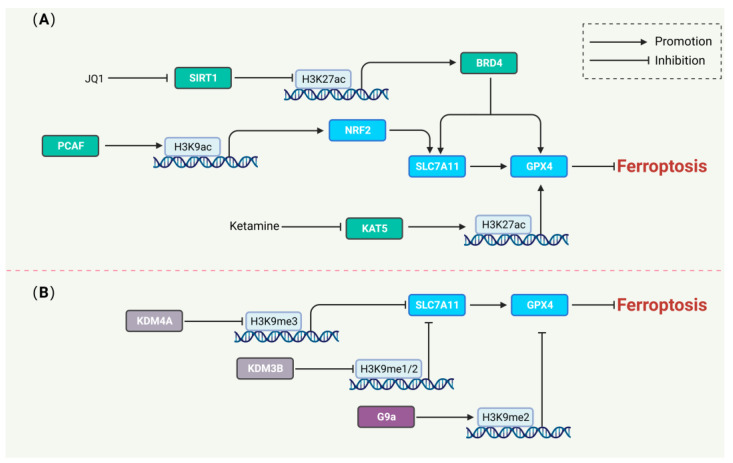
The regulation of ferroptosis by histone modification. (**A**) Histone acetylation in regulating ferroptosis. JQ1 and ketamine-induced ferroptosis by reducing the H3K27ac level of the BRD4 and GPX4 genes, respectively. PCAF inhibited ferroptosis by promoting the H3K27ac level of the NRF2 gene. (**B**) Histone methylation in regulating ferroptosis. KDM4A and KDM3B decrease the H3K9me3 and H3K9me1/2 levels of the SLC7A11 gene to inhibit ferroptosis. G9a promotes the H3K9me2 level of the GPX4 gene to induce ferroptosis.

Emerging studies suggest that that modifications of the core histone H2A, H2B, and H3 tails regulate gene expression by modulating interactions between histones and other nuclear proteins [128]. Previous studies have found that some specific types of cells are sensitive to ferroptosis due to differences in iron uptake and metabolic capacity, such as cancer stem-like cells (CSCs) and ovarian cancer tumor-initiating cells (TICs) [129,130,131,132]. Metal ions, including iron, can act as catalysts rather than cofactors to participate in epigenetic remodeling processes, and are deeply associated with development, inflammation, immune response, wound healing, and cancer progression [131,133]. Therefore, an in-depth analysis of the role of iron rather than ferroptosis in apparent remodeling is particularly urgent in subsequent studies. In addition, the function of iron to accelerate the ferroptosis process independent of the Fenton reaction and the function of the enzyme’s cofactors is also worthy of further study (Figure 4).

### 6.1. Histone Acetylation and Ferroptosis

Histone acetylation increases the accessibility of DNA for transcription factors, and is often associated with transcriptional activation [134]. The transcription factor NRF2 recruits P300/CBP-associated factor (PCAF) to increase the H3K9ac levels of NRF2 to regulate ferroptosis in renal tubulointerstitial fibrosis [135]. Ketamine, an inhibitor of lysine acetyltransferase 5 (KAT5), inhibits GPX4 by decreasing the levels of H3K27ac, leading to the execution of ferroptosis in breast cancer [136]. JQ1, an inhibitor of bromodomain protein 4 (BRD4), suppressed BRD4 expression by activating the SIRT1-mediated histone deacetylation of BRD4. The impaired BRD4 downregulates GPX4, SLC7A11, and SLC3A2 expression in breast and lung cancer cell lines and promotes ferroptosis [137].

### 6.2. Histone Methylation and Ferroptosis

Histone methyltransferases mediate histone methylation and usually inhibit gene transcription. The methylation modification of histone H3 promotes ferroptosis by regulating negative regulators of ferroptosis. Histone-lysine N-methyltransferase 2 (EHMT2/G9a) has been reported to be an epigenetic regulator of neuronal susceptibility to inflammation. The inhibition of GPX4 transcription-triggering ferroptosis by G9a-catalyzed histone H3 lysine 9 (H3K9me2) demethylation is a potential therapeutic target against inflammation-induced neurodegenerative diseases [138]. Histone demethylases (HDMs), such as the lysine-specific demethylases KDM3B and KDM4A, are crucial in regulating histone methylation homeostasis. The histone H3K9 demethylase KDM3B was found to synergistically upregulate SLC7A11 expression by activating transcription factor 4 (ATF4) to suppress ferroptosis [139]. KDM4A can promote the SLC7A11 transcriptional repression of ferroptosis through H3K9me3 demethylation in the SLC7A11 promoter region [140].

## 7. Conclusions and Future Perspectives

Ferroptosis is a complex cellular process, and the selective induction of ferroptosis has been used as a potential therapeutic strategy for certain cancers. However, when ferroptosis inducers are administered intravenously, they indiscriminately attack cells throughout the body, potentially causing serious toxic side effects [141]. Genetic mutations in many cancers are associated with cellular sensitivity to ferroptosis, so these mutations can be utilized to induce ferroptosis, thereby killing the cancer cells [142]. In addition, given that different cell groups have different sensitivities to ferroptosis, such as CSCs and TICs [129,130,131,132], this also provides a broad clinical prospect for solving the problem that immune-tolerant cancer cells are not sensitive to traditional cell death pathways (such as apoptosis and necrosis). Recent advances in epigenetic modification research have provided novel insights into the molecular mechanisms of ferroptosis and suggest that ferroptosis plays a crucial role in tumor therapy and other diseases. This review summarizes several epigenetic mechanisms that regulate ferroptosis, such as DNA methylation, RNA methylation, non-coding RNAs, and histone modifications. The current mechanism of RNA methylation regulating ferroptosis is mainly focused on the classical SLC7A11-GPX4 axis, and RNA methylation modifications in non-classical ferroptosis regulatory mechanisms need to be further investigated. An increasing number of epigenetic modifications that induce or inhibit ferroptosis targets are being identified, and combinations of epigenetic drugs with other conventional therapies are used in preclinical studies and clinical testing [12]. MiR-101-3p and E/M@FA-LP nanomedicines have promising antitumor effects in treating ferroptosis-associated tumors [108,143]. However, the modulation of ferroptosis through treatment with the same epigenetic modifications may show opposite effects in different tissues or diseases [12,50]. Epigenetic factors involve the development and progress of cancer and other diseases by regulating ferroptosis-associated genes. Still, the extent of the influence of ferroptosis on the development of cancer and other diseases remains unclear. There is an urgent need for a profound understanding of the molecular mechanisms of ferroptosis and its role in cancer and other diseases. Research on the regulation of epigenetic modifications in ferroptosis is still in its infancy. We must also find the significant epigenetic factors affecting ferroptosis to provide new therapeutic strategies for various diseases. In conclusion, the critical role of ferroptosis in cancer and other diseases provides a new idea for the study of therapeutic options for cancer and other diseases. More information on the regulatory mechanisms by which epigenetic modifications affect ferroptosis and their roles in cancer and other diseases will ensure that we better understand the pathogenesis of these diseases and use this as a basis for proposing new therapeutic modalities, which is a matter of urgency for us to address.

## Figures and Tables

**Table 1:** Comparison of ferroptosis in representative taxa.

Gene/Metabolite	Humans	Mice	Plants	Yeast	Bacteria
GPX4	Inhibitor [3]	Inhibitor	Inhibitor [14]	Presence of homologue [15]	Not found [16]
SLC7A11	Inhibitor	Inhibitor	Unknown	Unknown	Unknown
Glutathione	Synthetic substrate for GPX4	Synthetic substrate for GPX4	Works with ascorbate to suppress ROS	Antioxidant	Antioxidant
PUFAs	Essential for ferroptosis	Essential for ferroptosis	Essential for ferroptosis	Driving ferroptosis in CoQ-deficient yeast [17]	Not essential for ferroptosis
ACSL4	Promoter	Promoter	Present but unclearly function	Unknown	Unknown
Lipid peroxidation	Detected in ferroptosis	Detected in ferroptosis	Detected in ferroptosis	Detected in ferroptosis	Not typically observed
Vitamin E	Inhibitor	Inhibitor	Produced by many plants	Unknown	Unknown
Vitamin K	Inhibitor [18]	Inhibitor	Produced by many plants	Unknown	Unknown

## Data Availability

Not applicable.
